# Successful Outcome and Biliary Drainage in an Infant with Concurrent Alpha-1-Antitrypsin Deficiency and Biliary Atresia

**DOI:** 10.1155/2017/9348461

**Published:** 2017-11-29

**Authors:** Andrew W. Wang, Kimberly Newton, Karen Kling

**Affiliations:** ^1^Department of Surgery, Naval Medical Center San Diego, San Diego, CA 92134, USA; ^2^Division of Pediatric Gastroenterology, Hepatology, and Nutrition, Rady Children's Hospital, San Diego, CA 92123, USA; ^3^Department of Pediatrics, School of Medicine, University of California, La Jolla, San Diego, CA 92093, USA; ^4^Department of Surgery, School of Medicine, University of California San Diego, La Jolla, San Diego, CA 92093, USA; ^5^Division of Pediatric Surgery, Rady Children's Hospital, San Diego, CA 92123, USA

## Abstract

We describe the rare instance of concomitant biliary atresia and alpha-1-antitrypsin deficiency and the first documented successful portoenterostomy in this scenario. The potential for dual pathology must be recognized and underscores that prompt diagnosis of biliary atresia, despite concomitant alpha-1-antitrypsin deficiency, is essential to afford potential longstanding native liver function.

## 1. Introduction

For infants presenting with direct (conjugated) hyperbilirubinemia, the differential diagnosis remains vast, encompassing a wide array of medical and surgical liver diseases. As such, multiple serologic, radiologic, and histologic tests are employed simultaneously to make the correct diagnosis. Biliary atresia (BA) is diagnosed in approximately 1 in 19,000 live births [1]; it is the second most common underlying diagnosis in infants with liver disease [2] and is the most common indication for liver transplantation in the pediatric population [3]. In the case of BA, timely diagnosis and surgical intervention are critical to optimize liver function and overall outcome. When BA is suspected based on the overall clinical picture, an intraoperative cholangiogram is performed followed by corrective portoenterostomy (Kasai procedure) once the diagnosis is confirmed. There is urgency in making the diagnosis because delay in surgical treatment is associated with progression to liver failure. In contrast, alpha-1-antitrypsin deficiency (A1AT) can be seen more frequently in up to 1 in 1500 people [4], and its hepatic manifestations can present as cholestasis in the newborn period with a virtually overlapping clinical profile as that of biliary atresia. However, its management does not involve urgent surgical reconstruction as in BA. Abnormal folding of the A1AT Z (or S) mutant protein leads to intracellular accumulation which leads to degradation mechanisms thought to trigger cellular injury and apoptosis [5], and treatment is supportive. We describe a patient who presented with both A1AT and BA in whom the diagnosis of BA was simultaneously made early enough to perform corrective portoenterostomy before progression of liver damage.

## 2. Case Report

**Figure 1:**
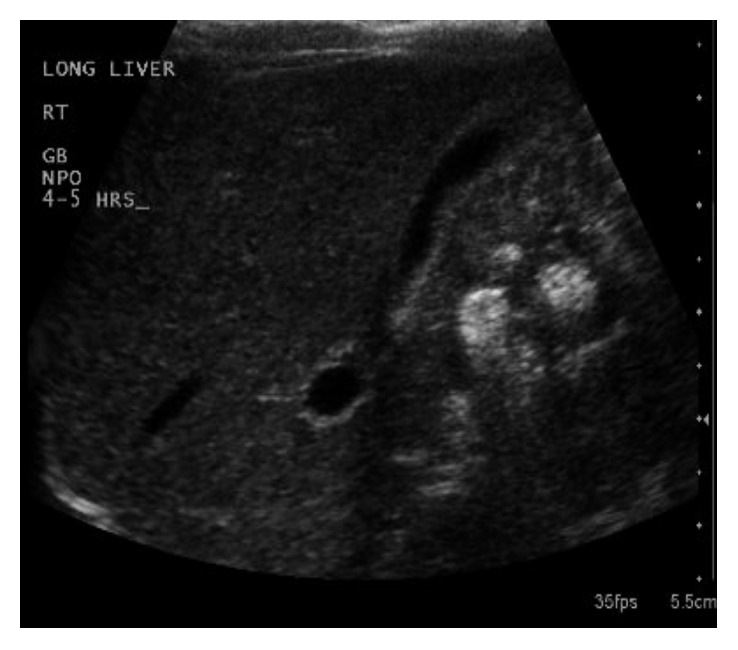
Abdominal ultrasound demonstrating a present gallbladder.

**Figure 2:**
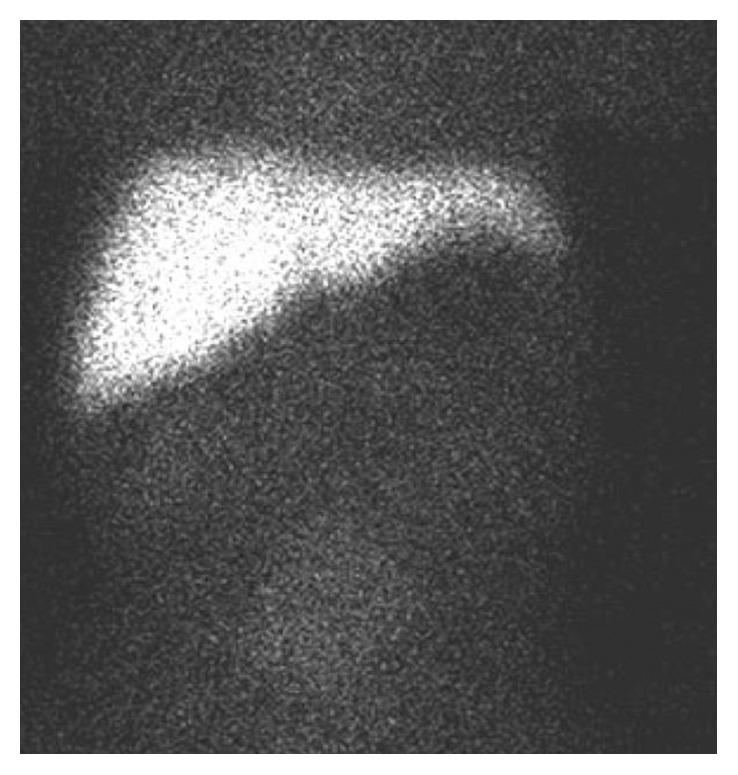
HIDA scan without excretion into the intestine.

An 11-day-old male infant presented for evaluation of direct (conjugated) hyperbilirubinemia. His prenatal studies were unremarkable; he was born at 38 weeks' gestation via Cesarean section due to breech presentation. The immediate postnatal course was pertinent for neonatal jaundice. A follow-up evaluation of serum bilirubin revealed a transition towards direct (conjugated) hyperbilirubinemia prompting referral to the gastroenterologist for further evaluation. The baby's parents noted his stools had become pale. His physical exam was remarkable for jaundice and hepatomegaly. Serum bilirubin was 4.7 mg/dL with a direct fraction of 3.2 mg/dL, and gamma glutamyl transferase was elevated to 600 U/L. Hematologic and coagulation studies were normal. Laboratory evaluation for metabolic causes of jaundice, including alpha-1-antitrypsin deficiency, cystic fibrosis, viral hepatitis, and glycogen storage disease, was performed, and while awaiting these results, an evaluation for BA and other surgical causes of conjugated hyperbilirubinemia was concurrently initiated. An abdominal ultrasound had no evidence of choledochal cyst and demonstrated the presence of a gallbladder but was equivocal for presence of the common bile duct (Figure 1). However, presence or absence of bile ducts on US does not provide conclusive evidence of BA as ductal structures seen on US may represent obliterated remnants of the biliary tree. A hepatobiliary iminodiacetic acid (HIDA) scan was performed to assess for biliary patency, and this failed to demonstrate excretion of the radioactive tracer into the duodenum (Figure 2). Percutaneous liver biopsy could not be obtained due to technical limitations.

**Figure 3:**
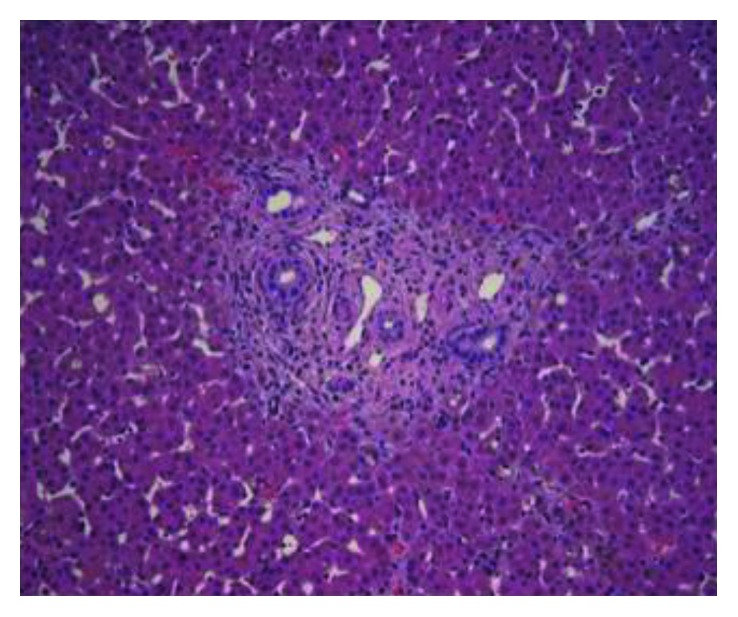
Intraoperative biopsy demonstrating bile duct proliferation.

**Figure 4:**
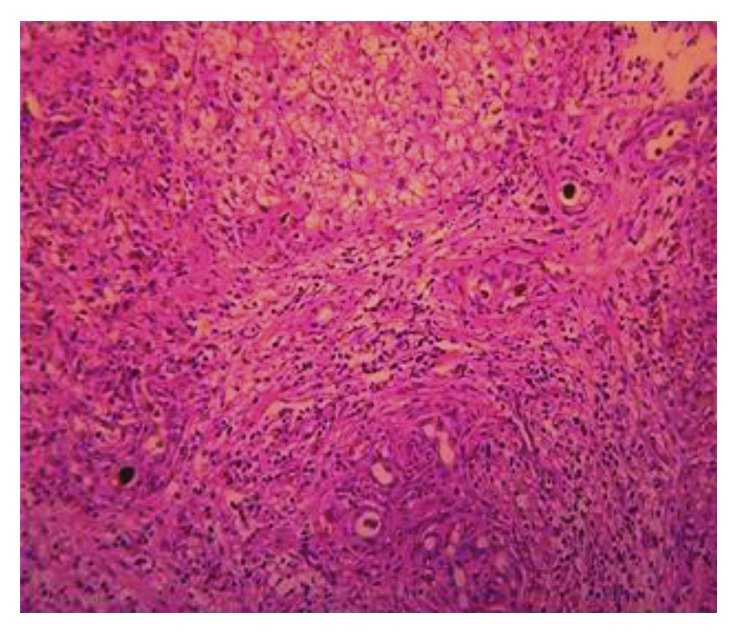
Intraoperative biopsy showing cholestasis and bile duct plugging.

**Figure 5:**
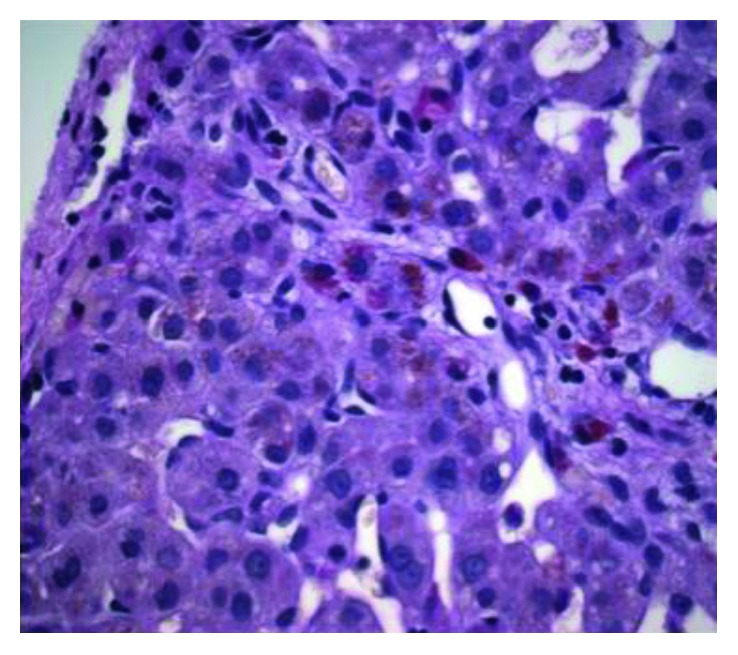
Intraoperative biopsy, hepatocytes with PAS-positive, diastase-resistant globules.

Given this convincing clinical picture specifically in conjunction with an abnormal HIDA scan, at 3 weeks of age, the patient underwent cholangiogram to evaluate for BA. Intraoperatively, only a hydropic gallbladder was identified consistent with nonpatency of at least part of the biliary tree. Although the subsequent cholangiogram demonstrated both the cystic and common bile ducts, the hepatic ducts were absent, suggesting that this proximal component of the biliary tree was atretic. Even with distal occlusion of the common bile duct and application of a significant amount of pressure instilling contrast in an attempt to distend and visualize a potentially small common hepatic duct, none was seen proving obstruction of the proximal biliary tree. While this is not the most typical finding associated with BA, patency of the gallbladder, cystic duct, and common bile duct with fibrosis of the hepatic ducts and porta can be seen [6]. While parts of the biliary system were patent, communication between the porta hepatitis and duodenum was absent consistent with BA; furthermore, dissection revealed the characteristic fibrosis of the hilar plate associated with BA. Thus, with a confirmed diagnosis of BA, portoenterostomy was performed without complication. Interestingly, during the Kasai reconstruction, the results of the serum alpha-1-antitrypsin analysis returned at 36 mg/dL, positive for alpha-1-antitrypsin disease. Liver biopsy routinely obtained intraoperatively ultimately confirmed both diagnoses. Histopathology demonstrated bile duct proliferation (Figure 3), cholestasis (Figure 4), chronic inflammation, and periportal fibrosis without bridging, consistent with the diagnosis of BA; additionally, hepatocytes with PAS-positive, diastase-resistant globules (Figure 5) were seen supporting the additional diagnosis of A1AT. Subsequent genotypic evaluation showed the PiZZ mutation, the most severe form of the disease. The patient recovered well postoperatively, and successful biliary drainage was achieved with his bilirubin and transaminases normalizing.

Over the next several years, the patient has continued to do well. Between 1 and 2 years of age, he had 3 very mild episodes of cholangitis which all resolved with antibiotics. The patient has not had any additional hospitalizations for cholangitis since that time and is now 8 years old. He has normal growth, near-normal liver function tests, and normal bilirubin, with normal coagulation and hematological parameters (direct bilirubin 0.1 mg/dL, AST 35 U/L, ALT 55 U/L, GGT 40 U/L, PTT 34, PT 11.7, PLT 310, and WBC 7.9). A recent abdominal ultrasound was unremarkable with normal hepatic echogenicity, without intrahepatic ductal dilatation, with normal spleen size, and with normal portal vein flow. An EGD performed in June 2016 was normal without evidence of varices. The patient is evaluated at regular intervals by pediatric hepatology and pulmonology. To date, there are no clinical signs of portal hypertension or lung disease.

## 3. Discussion

We present a patient with a dual diagnosis of A1AT and BA who was treated with a timely Kasai portoenterostomy with excellent outcome and 8-year survival to date with his native liver. Long-term prognosis for patients with A1AT deficiency who present with evidence of liver disease in infancy is reasonably favorable with approximately 80% manifesting no clinical evidence of chronic liver disease when followed into early adulthood [7]. In contrast, BA requires timely diagnosis and surgical treatment to achieve biliary drainage and prevent or delay progressive biliary disease. Currently, when appropriately identified and treated, biliary drainage is achieved in approximately 60% of cases leading to acceptable long-term survival as part of a multimodality approach to management including the eventual need for liver transplantation [8]. Delayed diagnosis of BA can lead to progressive cirrhosis necessitating primary liver transplantation [9]. The importance of prompt investigation of direct (conjugated) hyperbilirubinemia in infants presenting with acholic stools cannot be overemphasized.

Determining the underlying diagnosis in an infant with neonatal cholestasis can be challenging from both a clinical and a histopathological perspective. Biopsy results are not always straightforward as there can be substantial overlap among multiple hepatic diseases. Bile duct proliferation with plugging and fibrosis is characteristic of biliary atresia, but small-gauge percutaneous biopsies in infants may provide suboptimal tissue and insufficient portal tracts for adequate evaluation. At times, fibrosis and cholestasis without definitive evidence of proliferation and plugging is all that can be definitively demonstrated. Other diagnoses such as Alagille disease usually demonstrate bile duct paucity; however, this may not be apparent in very young infants. In A1AT, the characteristic PAS-positive globules can often be absent in very young infants leaving only nonspecific features of mild portal inflammation and fibrosis making definitive diagnosis elusive. Multiple diagnoses explaining neonatal cholestasis can have overlapping pathologic findings. A scenario lacking definitive diagnosis mandates cholangiogram to evaluate for biliary atresia and allow for timely surgical intervention if necessary. However, if a secure histopathologic diagnosis is established other than biliary atresia, such as A1AT, the workup usually ceases and treatment based on that diagnosis is begun. Biopsy in our case, if done preoperatively, most likely would have demonstrated PAS-positive, diastase-resistant globules and may or may not have demonstrated features of biliary atresia depending upon sample adequacy. Had the diagnosis of A1AT been made in the absence of convincing evidence of biliary obstruction, there would have likely been significant delay or complete failure to perform a cholangiogram and portoenterostomy, thereby resulting in increased liver damage and decreased or absent potential for biliary drainage. Although our usual practice is to obtain biopsy preoperatively, serendipitously, the inability to obtain biopsy, in this case, did not present us with a diagnosis of A1AT and therefore did not distract from prompt cholangiogram and portoenterostomy. Specifically, having considered other common confounding medical diagnoses (such as viral hepatitis, cystic fibrosis, TORCH infections, thyroid disease, pituitary issues, metabolic causes, cholesterol abnormalities, and bile acid diseases), given the time-sensitive nature of outcomes after portoenterostomy, we did not delay in obtaining cholangiogram. The cholangiogram did prove diagnostic of BA and made possible early portoenterostomy likely contributing to the good outcome for this patient. This underscores the importance of cholangiogram in determining the correct diagnosis and affording timely surgical reconstruction when appropriate.

Given the overlap among histopathologic findings in biliary atresia and other entities [10], this case illustrates that with sufficient clinical suspicion (as in our case of an infant with acholic stools, direct (conjugated) hyperbilirubinemia, and suggestive HIDA scan), despite other presumed diagnoses, cholangiogram, the gold standard for evaluation of biliary atresia, should still be pursued to definitively rule out BA. A HIDA scan without excretion despite a biopsy suggesting concomitant diagnoses such as A1AT or nonspecific neonatal giant cell hepatitis may still warrant cholangiogram. While operative cholangiogram does subject infants to anesthetic risks as well as a surgical scar, the operative morbidity is low. These risks must also be weighed in the context of potentially avoiding the lifelong morbidity of a missed diagnosis of BA and the missed opportunity for portoenterostomy. Additionally, in centers with the available resources and expertise, alternative techniques to operative cholangiography are becoming feasible including endoscopic retrograde cholangiopancreatography (ERCP) and percutaneous transhepatic cholecystocholangiography (PTC). These have been demonstrated to be useful and safe diagnostic alternatives to an open operative approach [10], potentially sparing some patients open operative procedures and a surgical scar. Endoscopic and percutaneous techniques may obviate operation for those in whom a patent ductal system can be demonstrated and may make cholangiography more palatable. These modalities were not available at our institution at the time of the evaluation of this infant. Ultimately, inability to complete a minimally invasive cholangiogram necessitates operative cholangiography.

More interestingly, this case report confirms that multiple etiologies can contribute to hepatic dysfunction; our patient had both A1AT and BA, and identifying the underlying diagnosis was essential in optimal management. This dual diagnosis is a very rare phenomenon; patients with BA almost universally have biopsy performed during their evaluation, yet there are only sparse case reports of synchronous A1AT and BA in the literature. Nord et al. and Tolaymat et al. previously report separate cases of patients found to have concomitant diagnoses of A1AT and BA. However, successful biliary drainage in this situation has not been reported to date and could not be achieved in either aforementioned patient; one patient underwent liver transplantation [11], and the other was being evaluated for future liver transplantation [12] by 9 months of age. Operative cholangiogram in patients known to have A1AT deficiency is not typically performed as the cholestasis associated with isolated A1AT is not mechanical. This is supported by a previous study of 11 patients with isolated A1AT deficiency evaluated with cholangiography that revealed normal extrahepatic biliary anatomy [13]. In isolated A1AT deficiency, this is a reasonable approach as the associated hepatic disease has an overall good prognosis. However, our case illustrates that there may exist concurrent diagnoses of BA and A1AT. The A1AT genotype in an infant may not yet be phenotypically expressed and may not therefore be the driving factor for the cholestatic picture; BA, as evidenced in this case, may be the predominant culprit. Thus, rarely, A1AT does not exclude BA, and securing the diagnosis of A1AT without ruling out BA may not be sufficient. This potential for synchronous diagnoses is an essential clinical situation to entertain because we demonstrate that Kasai portoenterostomy has the ability to preserve native liver in patients with this set of dual diagnoses.

## 4. Conclusions

We describe the very rare case of a patient who presented with both A1AT and BA and the first documented good outcome in the literature. He was successfully treated with Kasai portoenterostomy and currently survives with his native liver in situ and essentially normal liver function at age 8. This favorable outcome was achieved through early recognition of hyperbilirubinemia by the pediatrician accompanied by persistence to fully evaluate for BA. This was enhanced by the serendipity that evidence of a concurrent alpha-1-antitrypsin deficiency did not delay timely surgical cholangiography and biliary reconstruction. Our aim is not to suggest that one should abandon consideration of medical causes for hyperbilirubinemia or that all conjugated hyperbilirubinemia needs a cholangiogram, but rather to emphasize that all data must be considered and applied in the clinical context. Medical and surgical evaluations in these cases frequently proceed in a “shotgun” fashion due to the urgency of diagnosis; cholestasis should not be too quickly attributed to A1AT (a largely benign cause of cholestasis in infancy), when some elements of the workup suggest that biliary atresia (a more aggressive disease) may still be in play. This report illustrates that rarely dual diagnoses may exist. Despite what appears to be a nonsurgical diagnosis for hyperbilirubinemia, when the clinical scenario and HIDA scan do not completely rule out a diagnosis of BA, cholangiography should be pursued early enough that reconstruction can still effect a benefit. This is especially true as the recent advent of less invasive cholangiography techniques may alleviate some of the concern regarding committing an infant to open cholangiogram with a result negative for mechanical obstruction. A missed diagnosis of BA is a missed opportunity to surgically intervene and afford potential longstanding native liver function; this results in profoundly negative clinical consequences.
